# Movement coordination patterns between the foot joints during walking

**DOI:** 10.1186/s13047-017-0228-z

**Published:** 2017-10-30

**Authors:** John B. Arnold, Paolo Caravaggi, François Fraysse, Dominic Thewlis, Alberto Leardini

**Affiliations:** 1 Alliance for Research in Exercise, Nutrition and Activity, Sansom Institute for Health Research, University of South Australia, Adelaide, SA Australia; 2 Movement Analysis Laboratory, Istituto Ortopedico Rizzoli, via di Barbiano 1/10, 40136 Bologna, Italy; 3 Centre for Orthopaedics and Trauma Research, University of Adelaide, Adelaide, SA Australia

**Keywords:** Multi-segment foot kinematics, Rizzoli foot model, Walking, Foot joints, Coupling angle, Coordination pattern, Vector coding technique

## Abstract

**Background:**

In 3D gait analysis, kinematics of the foot joints are usually reported via isolated time histories of joint rotations and no information is provided on the relationship between rotations at different joints. The aim of this study was to identify movement coordination patterns in the foot during walking by expanding an existing vector coding technique according to an established multi-segment foot and ankle model. A graphical representation is also described to summarise the coordination patterns of joint rotations across multiple patients.

**Methods:**

Three-dimensional multi-segment foot kinematics were recorded in 13 adults during walking. A modified vector coding technique was used to identify coordination patterns between foot joints involving calcaneus, midfoot, metatarsus and hallux segments. According to the type and direction of joints rotations, these were classified as in-phase (same direction), anti-phase (opposite directions), proximal or distal joint dominant.

**Results:**

In early stance, 51 to 75% of walking trials showed proximal-phase coordination between foot joints comprising the calcaneus, midfoot and metatarsus. In-phase coordination was more prominent in late stance, reflecting synergy in the simultaneous inversion occurring at multiple foot joints. Conversely, a distal-phase coordination pattern was identified for sagittal plane motion of the ankle relative to the midtarsal joint, highlighting the critical role of arch shortening to locomotor function in push-off.

**Conclusions:**

This study has identified coordination patterns between movement of the calcaneus, midfoot, metatarsus and hallux by expanding an existing vector cording technique for assessing and classifying coordination patterns of foot joints rotations during walking. This approach provides a different perspective in the analysis of multi-segment foot kinematics, and may be used for the objective quantification of the alterations in foot joint coordination patterns due to lower limb pathologies or following injuries.

## Background

The foot is responsible for finalizing the force transmission between the lower limb and ground during locomotion. This is achieved by the complex kinematics and transfer of forces across foot and lower limb joints aimed at improving body propulsion and thus optimizing energy expenditure [1, 2]. Alternation between flexibility and rigidity of the foot joints during the stance phase allows the foot to address different mechanical requirements, such as adaptation to uneven terrains, absorption of the ground reaction forces, and assisting in forward progression [3, 4]. The complexity of foot dynamics, entailing the activation of intrinsic and extrinsic muscles across a number of joints to counteract the ground reaction forces throughout stance, is reflected by characteristic motion patterns of the foot joints [5, 6].

Temporal profiles of joint rotations are widely used in 3D gait analysis to report and assess foot motion during common motor tasks, but these are often analyzed in isolation thus preventing the observation of the complex kinematic interaction between adjacent joints. A vector coding technique [7] was devised to provide an easy representation and understanding of coordination patterns between body segments. The technique has been applied to the analysis of the coordination between foot segments during walking [8, 9] and for further classification into distinct coordination patterns [9–12]. While these studies have demonstrated that this technique can be applied to the analysis of the relationship between foot joints rotations, no comprehensive investigation on the coordination patterns between several foot joints, including the first metatarso-phalangeal joint, has thus far been reported. Moreover, coordination patterns between joint rotations across different planes and according to known anatomical and functional relationships in the foot, e.g. between ankle eversion and midtarsal joint dorsiflexion [13], has not been investigated.

The authors believe that the analysis of all joints is critical to gain a better understanding of the mechanisms controlling foot motion and deformation in gait. Moreover, although different methods are emerging for the presentation of movement coordination and variability [14], there is a lack of data pertaining specifically to average coordination patterns across multiple subjects and trials in the foot. Within this context, inter-joint coordination can be considered as the relationship in patterns of movement between joints. In particular, it is related to the timing and extent of rotation between joints involved in the same motor task. With a dynamical systems approach, movement coordination has been evaluated through continuous and discrete measures of relative phase [15, 16] and vector coding techniques [7].

In this study, a modified vector coding technique is applied to identify patterns of foot joint coordination during walking using an established multi-segment foot model incorporating the calcaneus, midfoot, metatarsus and hallux. This approach concurrently preserves the anatomical and clinical meaningfulness of the coordination patterns. An approach for visualising coordination patterns in the foot across multiple subjects is also presented.

## Methods

### Participants

Thirteen healthy adults were recruited in this study (5 M, 8 F; age 22.3 ± 2.7 years; height 1.74 ± 0.11 m; BMI 23.5 ± 3.6 kg/m^2^). All participants were free from lower limb pain and had no history of surgery, musculoskeletal or neurological conditions affecting locomotion or foot function. Individuals were taking part in a larger investigation on the effects of ageing on lower limb kinematics [17].

### Data collection

Reflective markers were placed on the right foot and leg according to the Rizzoli Foot Model [6, 18]. Accordingly, local reference frames are defined on the shank, calcaneus, midfoot, and metatarsus segments. Intersegmental rotations in the three anatomical planes between shank and calcaneus (ShCa), calcaneus and midfoot (CaMi), midfoot and metatarsus (MiMe), and calcaneus and metatarsus (CaMe) were calculated according to the convention established by Grood & Suntay (i.e. Joint Coordinate System) [19] and to the segmental reference frames described in the Rizzoli Foot Model [6, 18]. Sagittal- and transverse-plane motion of the hallux with respect to the metatarsus (MeHa) were also tracked [17]. For each joint coordinate system, rotation about the medio-lateral axis (x) was defined as plantarflexion/dorsiflexion, rotation about the antero-posterior axis (y) as inversion/eversion, and rotation about the longitudinal axis (z) as adduction/abduction. For the purpose of this manuscript, and for a better clinical interpretation, the ShCa joint will be also referred to as ankle joint, the CaMi as midtarsal joint, the MiMe as tarso-metatarsal joint, and MeHa as the 1st metatarso-phalangeal joint.

Twelve cameras (MX-F20, Vicon Metrics, UK) tracked markers’ trajectories at 100 Hz during level walking. Two floor-embedded platforms (9281B, Kistler Instrument Corp, Switzerland) recorded the ground reaction forces at 400 Hz. Walking speed was measured using two infrared photocells (Speed Light v2, Swift Performance Equipment, Australia). A static trial with each participant standing in relaxed bipedal upright posture was recorded to determine the joint angles neutral position. Each participant performed five walking trials at self-selected comfortable speed.

### Data processing

Marker trajectories were filtered using a 4th order low-pass Butterworth filter with a 6 Hz cut-off frequency [20]. Kinematic data were time normalized to stance phase duration using the ground reaction force data to detect stance events. Kinematic data processing was performed in Visual3D (v5.0, C-Motion, USA).

### Inter-joint coordination and vector coding technique

**Fig. 1:**
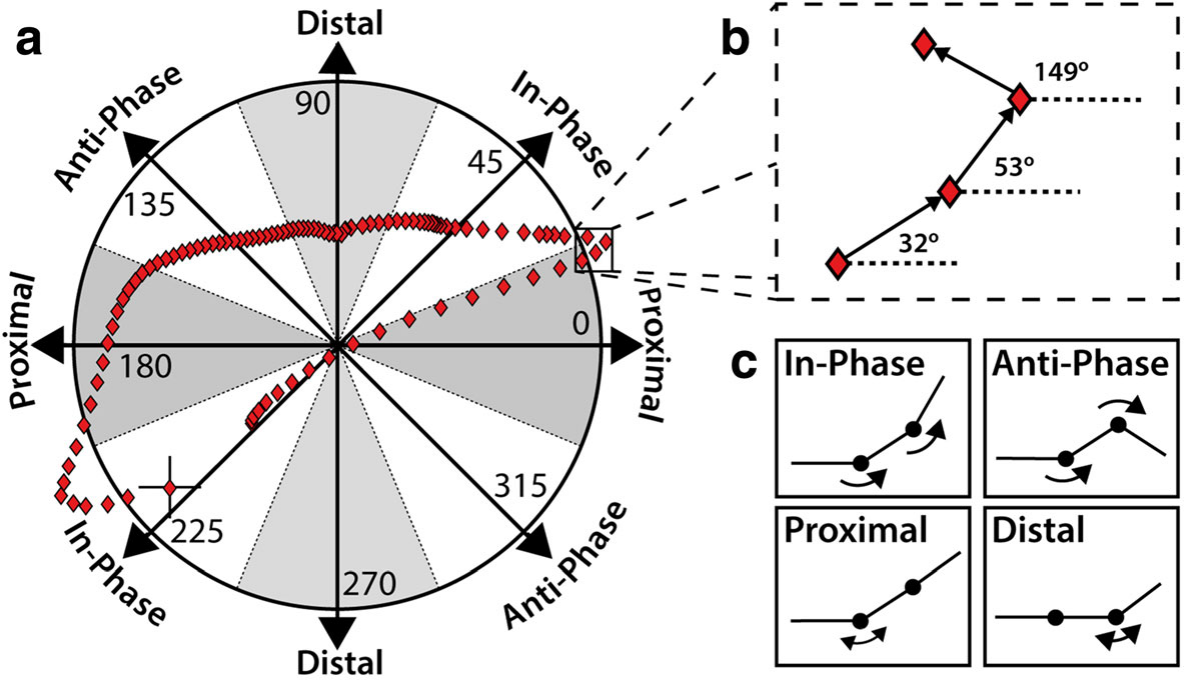
**a** Polar coordinate chart for the identification of coordination patterns between foot joints rotations. An exemplary trajectory of the coupling angle between ShCa (y) and CaMi (y) rotations is superimposed to the polar coordinate chart. **b** the frame-by-frame slope of the coupling angle is analysed to determine the coordination patterns. **c** Graphical representation of the four possible coordination patterns: in-phase; anti-phase; proximal, and distal

A modified vector coding technique, first described by Chang et al. [10] was implemented for the analysis of the coordination patterns in each of the following pairs of joint rotations:

1. CaMi (y) and ShCa (y);
2. CaMi (x) and ShCa (y);
3. MiMe (y) and ShCa (y);
4. MeHa (x) and ShCa (y);

where (*x)* and (*y)* refer to the sagittal- and coronal-plane joint rotations, respectively. These pairs of joint rotations were selected according to clinical interpretation of foot function and evidence from previous experimental studies [21–23]. Angle-angle plots were created for all trials from each participant (Fig. 1a) for the four pairs of rotations listed above. The frame-by-frame slope of the relationship between pairs of joints’ rotations was calculated (Fig. 1b) and will be further referred to as the coupling angle [10]. The coupling angles were classified according to four categories [10], defining four patterns of inter-joint coordination (Fig. 1c):

(i) *anti-phase*: coupling angle between 112.5–157.5 deg. or 292.5–337.5 deg., i.e. joints rotations have opposite directions;
(ii) *distal phase*: coupling angle between 67.5–112.5 deg. or 247.5–292.5 deg., i.e. distal joint rotation being more predominant than the proximal joint rotation;
(iii) *in-phase*: coupling angle between 22.5–67.5 deg. or 202.5–247.5 deg., i.e. joints rotations have the same direction;
(iv) *proximal phase*: coupling angle between 0 and 22.5 deg., 157.5–202.5 deg. or 337.5–360 deg., i.e. proximal joint rotation being more predominant than the distal joint rotation.

Since the direction of rotations in any anatomical plane is merely based on the adopted convention [6], anti-phase and in-phase coordination patterns can not be univocally assigned to pairs of joints rotations occurring in different anatomical planes (e.g. between CaMi (x) and ShCa (y)). Therefore, in this study, coordination patterns between joints rotations not occurring in the same anatomical plane will be conventionally defined as in-phase, if joints rotations are both positive or both negative according to the adopted convention (e.g. simultaneous CaMi dorsiflexion and ShCa eversion), and anti-phase if rotations have opposite directions in the adopted convention (e.g. simultaneous CaMi dorsiflexion and ShCa inversion).

The stance duration was divided into three periods: early (1–33% stance), middle (34–66% stance) and late stance (67–100% stance), approximately corresponding to the stance phases of loading response, mid-stance, and propulsion [10]. The duration (number of frames) of each of the four coordination patterns was determined for the three stance periods. Descriptive statistics (median and interquartile range) of the duration of each coordination pattern, in each stance period, were computed.

Moreover, the percentage of each coordination pattern across all samples was calculated and graphically represented via colour maps. This representation allows for an easy comprehension of the most predominant coordination pattern in each stance period. All computations were performed in MATLAB (R2015b, Mathworks, MA, USA).

## Results

**Fig. 2:**
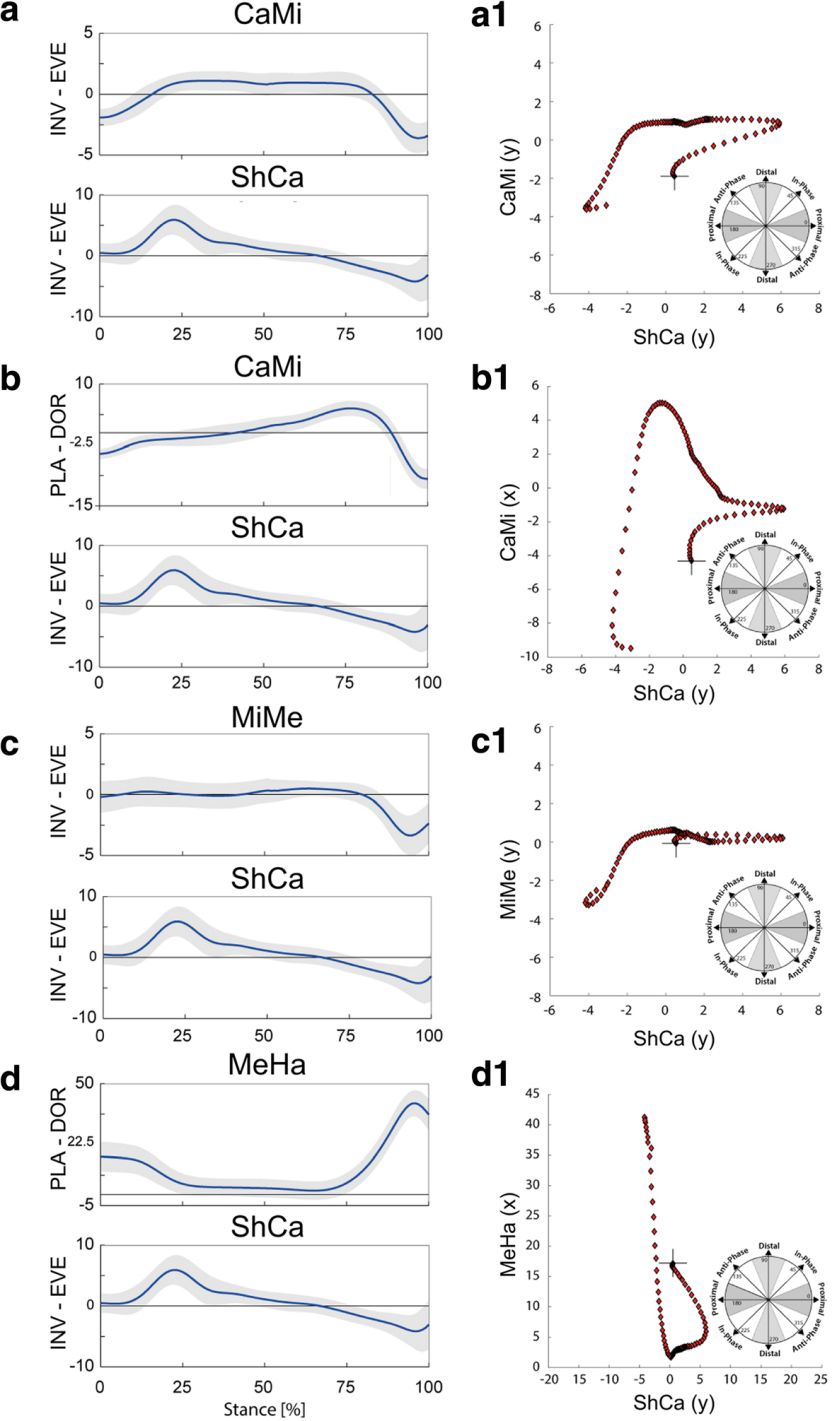
Left (**a**-**d**), mean temporal profiles of pairs of foot joints rotations (±1 standard deviation band) normalized to stance duration across all walking trials of all subjects. Right (**a1**-**d1**), angle-angle trajectories (i.e. coupling angle) for the pairs of joints rotations on the left. Where: ShCa (y) is the coronal-plane rotation of the midfoot with respect to the calcaneus; CaMi (x) and CaMi (y) are the sagittal and coronal-plane rotations of the midfoot with respect to the calcaneus; MiMe (y) is the coronal-plane rotation of the metatarsus with respect to the midfoot, and MeHa (x) is the sagittal plane rotation of the hallux with respect to the metatarsus. The trajectory initial point (0% stance) is highlighted by the ‘+’ symbol

The average walking speed across all walking trials was 1.35 ± 0.14 ms^−1^. The average time histories and angle-angle plots for the selected pairs of joints rotations are shown in Fig. 2 (a-d and a1-d1).

**Fig. 3:**
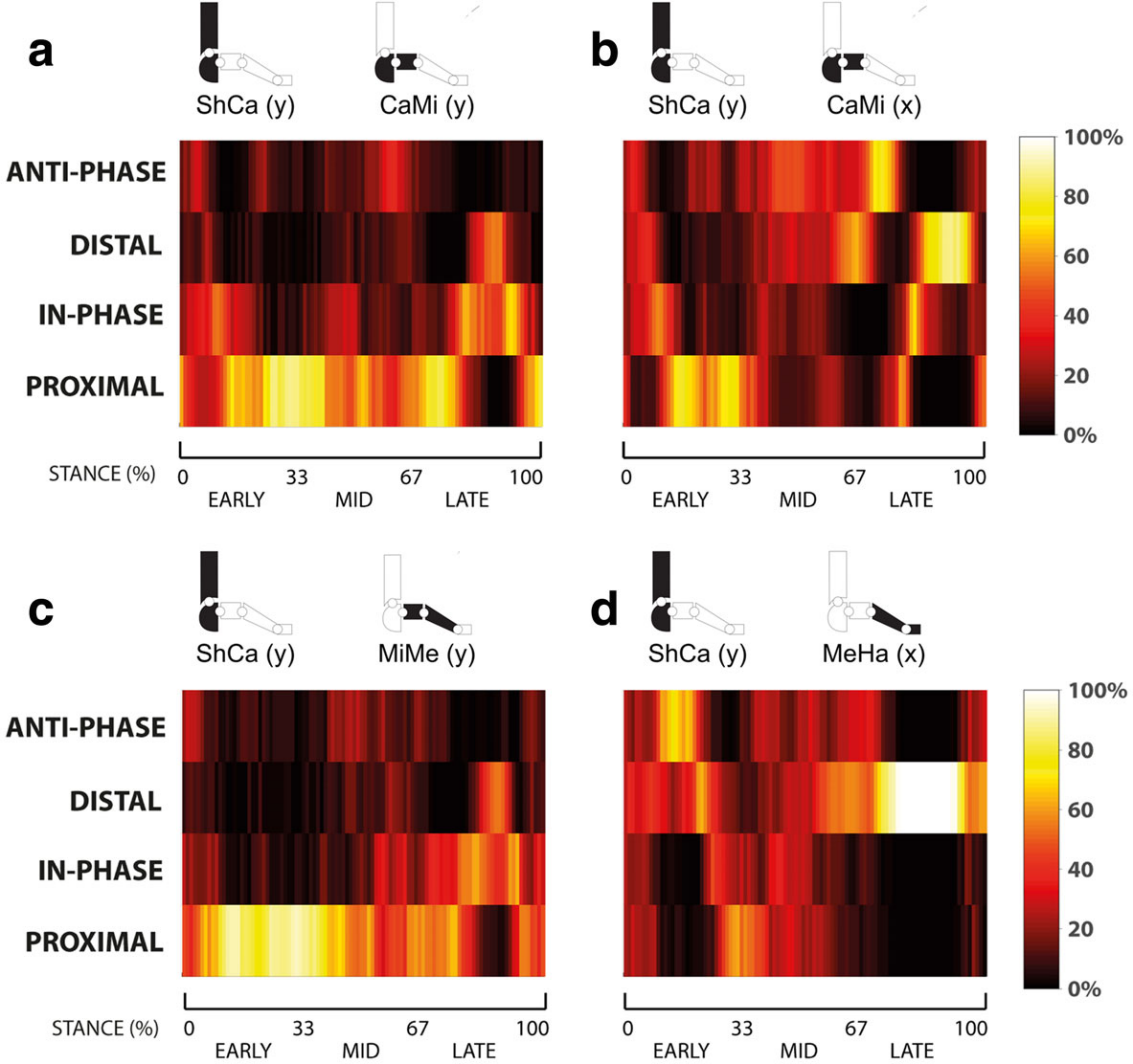
For each pair of joints rotations (**a**-**d**), color maps of the frame-by-frame percentage of walking trials in each coupling state during normalized stance duration. Where dark colors indicate lower percentages of walking trials in each coupling state and bright colors indicate larger percentages

Proximal phase coordination between ShCa (y) and CaMi (y) was predominant throughout early and middle stance (Fig. 3a and Table 1). In late stance, when the tarso-metatarsal joint also begins to invert, in-phase coordination becomes more predominant due to the simultaneous motion at both joints (Fig. 2a).

**Table 1:** Median frequency (# of frames) spent in each of the four coordination phases during early, middle and late stance. Values are presented as median [interquartile range]

	CaMi (y) - ShCa (y)	CaMi (x) - ShCa (y)	MiMe (y) - ShCa (y)	MeHa (x) – ShCa (y)
*Early* (1–33%)				
Anti-Phase	3 [3.5]	5 [4.5]	3 [4]	10.5 [5.5]
Distal	2 [2]	4.5 [4]	1 [2]	10 [7.5]
In-Phase	7 [6]	6 [6]	2 [4]	5 [5.5]
Proximal	19.5 [8]	17 [7.5]	25 [6]	6.5 [6]
*Mid (34–66%)*				
Anti-Phase	4.5 [7.5]	11 [11.5]	4 [5.5]	6.5 [6]
Distal	2 [4]	9 [9]	2 [4.5]	11 [12.5]
In-Phase	5 [4.5]	3.5 [7.5]	4 [6]	5.5 [8]
Proximal	19 [7.5]	5 [7.5]	20 [9.5]	7 [8]
*Late (67–100%)*				
Anti-Phase	1 [2]	8 [6.5]	1 [2.5]	3 [3]
Distal	5 [7]	16 [7.5]	4 [8]	28 [3.5]
In-Phase	12.5 [8]	6 [6.5]	14 [11]	1 [2]
Proximal	13 [5.5]	4 [3.5]	12 [11]	1 [1]

A shift from in-phase (simultaneous ankle eversion and midtarsal dorsiflexion) to proximal phase coordination in early stance was observed for coordination between ShCa (y) and CaMi (x) (Fig. 2b and Fig. 3b). A predominant anti-phase coordination was observed in middle stance and in the early part of late stance, due to the combined ankle inversion and midtarsal dorsiflexion. Subsequently, a shift to distal phase coordination pattern was detected due to rapid midtarsal plantarflexion (Fig. 3b).

Proximal phase coordination between ShCa (y) and MiMe (y) was found in early and middle stance (Fig. 3c and Table 1), primarily due to the limited mobility of the tarso-metatarsal joint relative to the more mobile ankle joint (Fig. 2c). Similar to coronal plane motion at the midtarsal joint, a shift to in-phase coordination was observed in late stance due to simultaneous inversion of tarso-metatarsal and ankle joints.

Anti-phase coordination between ShCa (y) and MeHa (x) was prominent in early stance, due to simultaneous ShCa eversion and MeHa plantarflexion (see Fig. 2d and Fig. 3d). This pattern shifts to proximal phase coordination as the ankle continues to evert once the hallux reaches the supporting surface. During late stance, a large gradient of 1st metatarso-phalangeal joint dorsiflexion compared to that of ankle inversion results in a strong distal phase coordination pattern (Table 1).

## Discussion

Foot joints mobility and coordination is achieved via interaction of intrinsic and extrinsic muscles acting across several joints under the constraint of soft tissues and ligaments. However, traditional kinematic analysis does not allow to capture the complexity of coordination between foot joint rotations. This study aimed at applying a modified vector coding technique for the analysis and representation of patterns of coordination between foot joints, comprising calcaneus, midfoot, metatarsus and hallux segments, in order to provide more insight into foot function during walking.

The main difference to previous analyses of foot joint coordination based on the vector coding technique is the inclusion of other foot joints, such as the midtarsal and first metatarsophalangeal joints, and the assessment of coordination patterns across different planes according to known anatomical and functional relationships in the foot. This study also used the proximal segment rather than the global coordinate system as the reference for joint rotations. From a biomechanical perspective, the present definition of proximal phase implies that while the proximal joint rotates the distal joint follows. The authors believe that this definition is more appropriate to describe the coordinative behaviour between foot joints as “proximal phase” and better reflects the idea of the proximal joint leading the motion of the distal one. With the previous definition such behaviour would be characterized as *in-phase,* which implies a synergistic mechanism [10].

Good qualitative consistency was found for kinematics of the foot joints with comparable results from previous studies [6, 17, 18, 24]. For coordinative patterns, similar to what was reported by Chang et al. [10], late stance in-phase coordination pattern between coronal-plane motion of the ankle and tarso-metatarsal joints was detected. The same coordination pattern was also found for the midtarsal joint. This suggests that late stance in-phase coordination may be present also in other foot joints. This seems in contrast to the classic view of simultaneous opposite rotations assisting foot stability during push-off [25]. Moreover, the presence of a predominantly distal coordination pattern, due to the rapid midtarsal joint plantarflexion in late stance, further reflects the complex interaction between joints in the foot across multiple planes of motion. Unlike what reported in previous studies [10], an increased frequency of proximal coordination between ankle and tarso-metatarsal joints was detected, associated to a less frequent distal coordination pattern (Table 1 and Fig. 3c). This difference is likely due to differences between foot models across studies, with less tarso-metatarsal motion probably biasing the pattern toward proximal coordination, as highlighted by the rather flat angle-angle relationship (Fig. 2 c1).

In addition to the intrinsic limitations of kinematic analysis based on skin markers, and to the relatively small sample size used here, it should also be pointed out that the vector coding technique is sensitive to small joint rotations and rotation velocities [10]. We attempted to mitigate this limitation by using kinematic measures from an established and reliable foot model [26], and by excluding further coupling relationships which could have been more susceptible to errors [24]. Methods for presenting detailed coordination profiles along with additional data on segmental dominancy and variability are also emerging [14]. Since this was the first study reporting coordination patterns using the vector coding technique for all joints within the Rizzoli Foot Model, we adopted an established technique specifically designed for foot analysis [10]. Future studies can benefit from using different techniques to reveal additional details about coordination of foot joint motion during walking and other activities [14].

## Conclusions

This study has identified and classified coordination patterns across a number of joints in the foot during walking. Identifying coordination patterns of foot joint motion offers a different perspective in the analysis of multi-segment foot kinematics, and may be used for the objective quantification of alterations in foot joint coordination patterns thus assisting in the clinical interpretation of foot and lower limb pathologies.

## Abbreviations

CaMi (x) and CaMi (y): sagittal- and coronal-plane rotations of calcaneus - midfoot joint
MeHa (x): sagittal-plane rotation of first metatarso-phalangeal joint
MiMe (y): coronal-plane rotation of midfoot-metatarsus joint
ShCa (y): coronal-plane rotation of shank - calcaneus joint
